# Molecular Mechanisms Associated with Plant Tolerance Under Abiotic Stress—Second Edition

**DOI:** 10.3390/plants15060852

**Published:** 2026-03-10

**Authors:** Emilia Apostolova

**Affiliations:** Institute of Biophysics and Biomedical Engineering, Bulgarian Academy of Sciences, Acad. G. Bonchev Str., Bl. 21, 1113 Sofia, Bulgaria; emya@bio21.bas.bg or emilia.apostolova@gmail.com

Abiotic environmental stressors are significantly exacerbated by climate change and negatively impact plant growth, development, and productivity [1]. In natural conditions, plants are exposed to combinations of abiotic stresses that require coordinated and integrated adaptive responses. A common consequence of abiotic stress is increased production of reactive oxygen species (ROS), which can damage DNA, pigments, proteins, and other essential cell components, altering plant physiology [2]. Simultaneously, ROS are essential for the detection of biotic and abiotic stress, the integration of various environmental signals, and the activation of stress-response networks [3]. To survive harsh environments, plants undergo numerous morphological and metabolic changes, which vary not only among species but also among genotypes within the same species [4,5,6]. Studying the molecular mechanisms underlying plants’ tolerance of abiotic stress is essential for improving agricultural productivity and ensuring food security in the face of climate change. Identifying key stress-response genes, transcription factors, and metabolic pathways is important for developing resilient crop varieties capable of withstanding environmental challenges, enhancing food security, and supporting sustainable agriculture under increasing abiotic stress conditions.

Photosynthesis, the process whereby light is converted into chemical energy, is highly sensitive to climate change [7]. The light-harvesting complex of photosystem II (LHCII) plays a central role in regulating photosynthetic efficiency and can exhibit dynamic structural organization, forming monomers, dimers, trimers, and aggregates, depending on light intensity [8]. The dynamic organization of LHCII is essential for optimizing light-energy capture and efficient photosynthesis [9]. A recent study revealed that variations in LHCII organization significantly influence energy absorption, the density of the reaction centers of photosystem II (PSII), the reoxidation of Q_A_−, and electron transport efficiency [10]. The authors showed that increased LHCII oligomerization increased the number of open reaction centers of photosystem II (PSII) and their excitation efficiency and improved photosynthetic performance under physiological conditions [10]. Importantly, differences in LHCII organization have also been linked to plants’ sensitivity to drought stress [11]. Plants exhibiting higher degrees of LHCII oligomerization show reduced inhibition of PSII photochemistry, electron transport, and photosynthetic rates under stress [11].

Plants have evolved various defense mechanisms, with diverse signal transduction pathways playing a central role. Among these pathways, calcium ions (Ca^2+^) act as second messengers, mediating a wide spectrum of environmental and developmental signals [12]. Calcium-dependent protein kinases (CDPKs) are key Ca^2+^ sensors in plants and mediate responses to abiotic stresses through phosphorylation signaling. A recent study analyzing the CDPK gene family in the halophyte *Nitraria sibirica* revealed its structural diversity, evolutionary conservation, and multifaceted regulatory roles in abiotic stress adaptation. The study showed a potential regulatory module involving MYB transcription factors and calcium-dependent protein kinases, offering new insights into the molecular mechanisms underlying extreme salt tolerance [13].

Studies have also demonstrated that ZF-HD transcription factors play critical roles in plant growth, development, and responses to abiotic stresses among various species, including eggplant, quinoa, Arabidopsis, and rice [14,15,16,17]. The authors of a recent study conducted a genome-wide identification and expression analysis exploring the primary functional characteristics of the ZF-HD gene family in melon (*Cucumis melo* L.) under salt stress [18]. The authors identified 13 ZF-HD genes in the melon genome using bioinformatics approaches and performed a systematic, comprehensive analysis. These genes were classified into ZHD subfamilies I–IV and the MIF subfamily. Analysis of the evolutionary features and functional characteristics of the CmZF-HD family in response to salt stress identified CmZHD8 as a key candidate gene for stress resistance in melon [18].

In recent years, it has been revealed that DUF proteins are involved in important physiological and biochemical processes in plants as well as in responses to biotic and abiotic stresses. The DUF4228 family, which exists exclusively in plants, was renamed to PADRE (Pedigree-Aware Distant-Relationship Estimation) based on its known functional characteristics. In *Arabidopsis thaliana*, Chang et al. [19] revealed that AtPADRE13 is associated with plant responses to abiotic stress and may also participate in abscisic acid (ABA)-mediated signaling pathways. Overexpression of AtPADRE13 in *Arabidopsis thaliana* resulted in downregulation of positive regulatory genes under salt stress conditions [19]. The data further showed that in overexpression lines, malondialdehyde (MDA) levels increased, while antioxidant enzyme activity decreased.

Plants’ growth and development can be significantly affected by alkali stress. This type of stress involves not only osmotic stress and ion toxicity but also damage caused by high pH levels [20,21]. Alkali stress has a more severe impact on plants than salt stress [22]. It leads to enhanced ROS production and activation of the oxidant defense systems, resulting in increased superoxide dismutase (SOD), peroxidase (POD), catalase (CAT), and peroxidase (APX) activity. Moreover, many genes involved in photosynthesis are significantly upregulated under alkali stress. A recent study on *Sorghum bicolor* L. genotypes with different levels of alkaline resistance revealed that the MAPK (Mitogen-Activated Protein Kinase) signaling pathway and plant hormone signal transduction pathways are involved in alkali stress responses. Several transcription factor families, including bHLHs, ERFs, NACs, and MYBs, were differentially expressed in response to alkali stress conditions [23].

Plants have developed multiple adaptive mechanisms to alleviate the effects of abiotic stresses. A recent study highlighted the role of non-CG DNA methylation in Arabidopsis under cadmium stress [24]. The authors compared the effects of cadmium stress on the root apical meristem in wild-type and *ddc* triple mutants (deficient in DRM 1, DRM 2, and CMT3). Under cadmium stress, auxin signaling was maintained and ROS accumulation was reduced in the *ddc* mutant compared to the wild type. The authors concluded that DNA methylation shapes the hormonal and oxidative landscape of the root meristem, thereby promoting developmental resilience under heavy-metal exposure [24].

Environmental temperature is the most important factor influencing plant growth, development, and yield. High temperatures induce significant changes in key physiological and molecular mechanisms in plants [25]. Under high-temperature conditions, levels of ROS increase. Although ROS in excessive quantities can damage essential cellular components, they also function as signaling molecules that induce the synthesis of protective compounds, thereby enhancing plant tolerance. A recent study revealed that BPM (BTB/POZ-MATH) proteins regulate plant responses to heat stress in *Arabidopsis thaliana* [26]. The equilibrium between stress signaling and cellular homeostasis may be upset by BPM1 overexpression, while reduced expression of several BPM genes may enable a more sustained yet regulated activation of heat-responsive pathways [26]. A study on broccoli (*Brassica oleracea*) demonstrated that the effects of high temperatures vary depending on a plant’s developmental state [27]. It was shown that developmental stage and organ maturity play a dominant role in shaping the metabolic profile of broccoli, although temperature can modulate specific metabolic responses at each stage. The most substantial changes observed in mature broccoli heads included increased proline content, a higher chlorophyll a/b ratio, and enhanced DPPH radical-scavenging activity. Under high-temperature conditions, mature broccoli leaves exhibited lower susceptibility than seedlings [27].

Drought stress is an environmental abiotic stress that greatly influences plant growth and yield. The review by Michalak et al. [28] summarized the molecular mechanisms and experimental approaches used to understand plant drought responses. The authors highlighted key regulatory components, specifically the ABA and ROS signaling pathways, as well as the role of osmoprotectant accumulation and stomatal regulation under drought stress [28]. Melatonin, a non-toxic agricultural biostimulant, plays a critical role in mitigating environmental stresses such as drought, salinity, high temperatures, and heavy-metal toxicity. In plants, it serves as a natural antioxidant with growth-regulating properties. A recent study demonstrated that melatonin application alleviates drought-induced damage to the photosynthetic apparatus and supports recovery after stress [29].
